# Maternal and Neonatal Outcomes Based on Changes in Glycosylated Hemoglobin Levels During First and Second Trimesters of Pregnancy in Women with Pregestational Diabetes: Multicenter, Retrospective Cohort Study in South Korea

**DOI:** 10.3390/life14121575

**Published:** 2024-12-01

**Authors:** Mi Ju Kim, Suyeon Park, Sooran Choi, Subeen Hong, Ji-Hee Sung, Hyun-Joo Seol, Joon Ho Lee, Seung Cheol Kim, Sae-Kyoung Choi, Ji Young Kwon, Seung Mi Lee, Se Jin Lee, Han-Sung Hwang, Gi Su Lee, Hyun Soo Park, Soo-Jeong Lee, Geum Joon Cho, Jin-Gon Bae, Won Joon Seong, Hyun Sun Ko

**Affiliations:** 1 Department of Obstetrics and Gynecology, School of Medicine, Kyungpook National University, Daegu 41944, Republic of Korea; ties1004@naver.com; 2 Department of Obstetrics and Gynecology, Inha University, Incheon 22212, Republic of Korea; hi0438@naver.com (S.P.); csran@inha.ac.kr (S.C.); 3 Department of Obstetrics and Gynecology, Incheon St. Mary’s Hospital, The Catholic University of Korea, Seoul 06591, Republic of Korea; unihsy@naver.com (S.H.); obgysk@catholic.ac.kr (S.-K.C.); jiyoungk@catholic.ac.kr (J.Y.K.); 4 Department of Obstetrics and Gynecology, Sungkyunkwan University, Seoul 03063, Republic of Korea; jihee.sung@samsung.com; 5 Department of Obstetrics and Gynecology, Kyung Hee University, Seoul 02447, Republic of Korea; seolhj@khu.ac.kr; 6 Department of Obstetrics and Gynecology, Yonsei University Health System, Yonsei University, Seoul 03722, Republic of Korea; doctor-joon@hanmail.net; 7 Department of Obstetrics and Gynecology, Pusan National University, Busan 46241, Republic of Korea; ksch0127@naver.com; 8 Department of Obstetrics and Gynecology, Seoul National University, Seoul 08826, Republic of Korea; lbsm@snu.ac.kr; 9 Department of Obstetrics and Gynecology, Kangwon National University, Gangwon 24341, Republic of Korea; 23wls@naver.com; 10 Department of Obstetrics and Gynecology, Konkuk University, Seoul 05030, Republic of Korea; 20080251@kuh.ac.kr; 11 Department of Obstetrics and Gynecology, School of Medicine, Keimyung University, Daegu 42601, Republic of Korea; cllgs315@naver.com (G.S.L.); gonmd@dsmc.or.kr (J.-G.B.); 12 Family Medicine Residency, Providence St. Joseph Eureka Hospital, Eureka, CA 95501, USA; hsparkmd@gmail.com; 13 Department of Obstetrics and Gynecology, College of Medicine, University of Ulsan, Ulsan 44610, Republic of Korea; exsjlee@uuh.ulsan.kr; 14 Department of Obstetrics and Gynecology, Korea University, Seoul 02841, Republic of Korea; md_cho@hanmail.net; 15 Department of Obstetrics and Gynecology, Seoul St. Mary’s Hospital, College of Medicine, The Catholic University of Korea, Seoul 06591, Republic of Korea

**Keywords:** first trimester, glycosylated hemoglobin, maternal outcomes, neonatal outcomes, pregestational diabetes, second trimester

## Abstract

This study compared glycosylated hemoglobin (HbA1c) levels in the first and second trimesters of pregnancy and assessed maternal and neonatal outcomes according to HbA1c variations among women with pregestational diabetes. This retrospective, multicenter Korean study involved mothers with diabetes who had given birth in 17 hospitals. A total of 292 women were divided into three groups based on HbA1c levels during the first and second trimesters: women with HbA1c levels maintained at <6.5% (well-controlled [WC] group); women with HbA1c ≥ 6.5% (poorly-controlled [PC] group); and women with HbA1c ≥ 6.5% in the first trimester but <6.5% in the second trimester (improved-control [IC] group). The PC group had the highest pregnancy-associated hypertension (PAH) incidence, while the incidence did not significantly differ between the WC and IC groups. The receiver operating characteristic (ROC) curve indicated that HbA1c in the second trimester could predict PAH with a cut-off value of 5.7%. The PC versus WC versus IC group showed statistically significantly higher neonatal birthweight and significantly higher rates of large for gestational age (LGA); however, those were not significantly different between the WC and IC groups. HbA1c levels in the second trimester could predict LGA, with a cut-off value of 5.4%. Therefore, the second trimester HbA1c levels were significantly associated with both maternal and neonatal outcomes.

## 1. Introduction

Women diagnosed with type 1 or 2 diabetes before pregnancy (pregestational diabetes) account for about 1–2% of all pregnancies (approximately 14.9 million women), but recently, the frequency of pregestational diabetes has been increasing due to an obesity-related rise in type 2 diabetes [1]. Pregestational diabetes also affects women without a history of type 1 or 2 diabetes before pregnancy, but with glycosylated hemoglobin (HbA1c) ≥ 6.5% in the first trimester or early second trimester, a fasting glucose level ≥ 126 mg/dL, or a 2-h glucose level ≥ 200 mg/dL after a 75 g oral glucose tolerance test [2]. Pregestational diabetes is associated with maternal morbidity during pregnancy and various complications. Like mothers with uncontrolled gestational diabetes, pregestational diabetes is relatively common in cases of cesarean section, preterm labor due to polyhydramnios, and pregnancy-associated hypertension (PAH). The frequency of these complications varies depending on glucose control [2]. Diabetes is associated with perinatal mortality and morbidity, thus increasing the incidence of congenital anomalies, abortion, stillbirth, respiratory distress syndrome (RDS), and macrosomia, which, in turn, affect the short- and long-term prognosis of newborns [2]. In particular, macrosomia has been reported to have an incidence about twice as high in patients with pregestational diabetes compared to non-diabetic mothers, with one study reporting a frequency of 11.8%. It has been confirmed that the incidence of shoulder dystocia during delivery is more than twice as high with macrosomia, which is believed to have a significant impact not only on the short-term but also on the long-term prognosis of the newborn [3]. Therefore, controlling blood sugar properly during pregnancy is crucial to reducing the frequency of related complications [4].

HbA1c measurement is widely used as a screening test to assess the level of blood sugar control during pregnancy [5]. HbA1c indicates the irreversible attachment of glucose to hemoglobin and reflects blood sugar levels over approximately 2–3 months as hemoglobin is destroyed and regenerated every 2–3 months. Further, HbA1c is relatively stable, shows little short-term variation due to meals, and can be easily checked without fasting [6]. The general cut-off value for HbA1c is set at 6.5%, with the diagnosis of diabetes established at values above this level [7]. However, this diagnostic criterion is used in nonpregnant women; hence, it may be unreasonable to apply it to pregnant women. In early pregnancy, the lifespan of erythrocytes and blood glucose concentration decrease, reducing HbA1c levels. A recent meta-analysis reported that HbA1c < 5.7% during early pregnancy indicates better pregnancy-related outcomes [6]. However, there may be differences by race. To the best of our knowledge, few studies have serially checked HbA1c values during the first and second trimesters of pregnancy in women with pregestational diabetes and compared maternal and newborn outcomes, especially in Asian populations [8,9].

Therefore, this study aimed to measure HbA1c levels in Korean women with pregestational diabetes during the first and second trimesters of pregnancy, compare the changes, evaluate the outcomes for mothers and newborns, and propose a target HbA1c value that could reduce the incidence of complications.

## 2. Materials and Methods

### 2.1. Study Design and Study Population

This study was a retrospective cohort study targeting patients with pregestational diabetes who completed prenatal examinations serially during the first trimester of pregnancy until delivery at 17 tertiary hospitals in South Korea from January 2010 to December 2023. Of the 747 mothers, we excluded women who had chronic hypertension, multiple pregnancies, chromosomal abnormalities, non-viable preterm birth (<24 weeks), or who did not have HbA1c levels (Bio Rad, Richmond, CA, USA) recorded in the first and second trimester.

**Figure 1:**
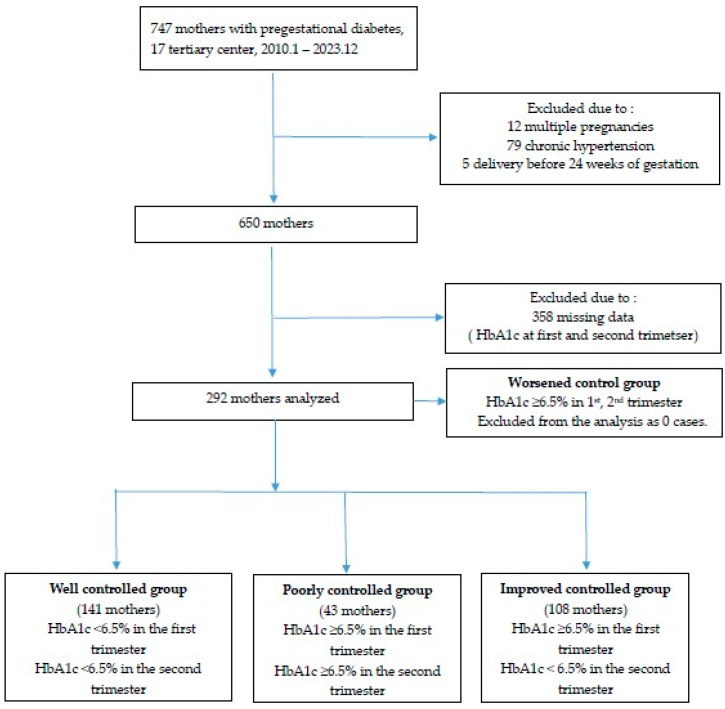
Flowchart of enrollment for study participation.

In women with HbA1c levels available during the first and second trimesters of pregnancy, glycemic control status was evaluated based on an HbA1c cut-off value of 6.5%. Women with HbA1c < 6.5% in both the first and second trimesters were classed as the well-controlled (WC) group; women with HbA1c ≥ 6.5% in both trimesters were classed as the poorly-controlled (PC) group; and women with HbA1c ≥ 6.5% in the first trimester, but <6.5% in the second trimester were classed as the improved- control (IC) group. Additionally, women with HbA1c < 6.5% in the first trimester but ≥6.5% in the second trimester were classed as the deterioration-control (DC) group (Figure 1); however, the DC group was excluded from the analysis because there were no cases of deterioration.

### 2.2. Study Assessments

We analyzed maternal characteristics such as age, parity, pregnancy method, underlying disease, and body mass index (BMI) among the three groups and assessed average HbA1c values for each trimester. Maternal underlying diseases before pregnancy included hyperthyroidism, hypothyroidism, thyroid cancer, chronic kidney disease, major depression, and respiratory diseases such as asthma. In the second and third trimesters of pregnancy, we examined the expected fetal weight and abdominal circumference measured by ultrasound as well as the presence of polyhydramnios or oligohydramnios. Complications during pregnancy were evaluated based on hospitalization and reasons for hospitalization, if applicable, such as threatened preterm labor, preterm premature membrane rupture, incompetent cervix, PAH, and uncontrolled diabetes. Threatened preterm labor was characterized by the need for inpatient treatment before 37 weeks of gestation, which was indicated by the presence of regular uterine contractions with cervical change.

### 2.3. Study Outcomes

A composite adverse maternal outcome during pregnancy was defined as any of the following: hospitalization during pregnancy, or PAH, diabetes-related retinopathy, nephropathy, neuropathy, ketoacidosis, or infection during pregnancy. Obstetric complications at delivery included fetal death in utero (FDIU), preterm birth, shoulder dystocia, genital tract laceration, clinical chorioamnionitis, postpartum endometritis, wound infection or dehiscence, and postpartum bleeding. Shoulder dystocia was defined as failure of a shoulder after downward movement, as identified by medical record, or requiring maneuver (e.g., McRobert maneuver) and gentle downward traction on the fetal head after delivery [10]. Genital tract laceration was defined as any event, including third or fourth-degree laceration, vaginal wall laceration, or urethral or bladder injury. Postpartum endometritis refers to infection of the endometrium, myometrium, and surrounding uterine tissue after delivery, occurring in febrile cases without any other obvious cause of the fever [11,12]. Clinical chorioamnionitis was diagnosed using one or more of the following criteria: maternal fever ≥ 38 °C, maternal or fetal tachycardia, or maternal white blood cell count ≥ 15,000/mm^3^. Postpartum bleeding was defined as significant bleeding of >1 L or necessitating transfusion due to anemia following delivery. A composite obstetric adverse outcome was defined as any one of the following: FDIU, preterm birth, shoulder dystocia, genital tract laceration, clinical chorioamnionitis, postpartum endometritis, wound infection or dehiscence, or postpartum bleeding. The neonatal outcomes analyzed included sex, gestational age at delivery, neonatal birth weight, 1 and 5 min Apgar scores, and the rates of small for gestational age (SGA), large for gestational age (LGA), admission to a neonatal intensive care unit (NICU), congenital anomalies, and RDS. SGA was defined as weight below the 10th percentile, adjusted for gestational age and sex, while LGA was defined as weight above the 90th percentile [13]. Composite neonatal morbidity was defined as the presence of ≥1 of the following during the neonatal period: admission to the NICU; neonatal death within 72 h after birth; Apgar score < 7 at 1 min or 5 min; congenital anomalies; umbilical cord pH < 7.1; hypoglycemia (blood sugar < 40 mg/dL), hyperbilirubinemia (>15 mg/dL); hypocalcemia (<7–8 mg/dL); polycythemia (>22 mg/dL); RDS; sepsis; cardiomyopathy; pulmonary hypertension; or seizures.

### 2.4. Statistical Analyses

Statistical analyses were performed using SPSS software version 12.0 (IBM Corporation; Armonk, NY, USA). Three groups were compared: WC, PC, and IC. Continuous variables were analyzed using the Student’s *t*-test and were expressed as mean ± standard deviation (SD), whereas categoric variables were analyzed using the Fisher’s extract test and expressed as percentages. A *p*-value < 0.05 indicated statistical significance. A multivariate analysis was conducted for each maternal or neonatal complication to calculate the odds ratio (OR). A receiver operating characteristic (ROC) curve was drawn to present the HbA1c cut-off value for each complication.

### 2.5. Ethics Statement

The study was conducted in accordance with the guidelines of the Declaration of Helsinki and was approved by the Institutional Review Boards (IRBs) of all 17 centers. The IRB of Kyungpook National University Hospital and of each participating hospital reviewed and approved this study (IRB no. 2023-03-011-011). Informed consent was waived because of the retrospective nature of the study.

## 3. Results

Data for 292 mothers with pregestational diabetes and HbA1c values available during the first and second trimesters of pregnancy were analyzed. Within this cohort, 141, 108, and 43 mothers were classed into the WC, IC, and PC groups, respectively (Table 1).

Maternal baseline characteristics, such as age, parity, and artificial reproductive technique usage, did not show significant differences among the three groups. However, BMI just before delivery was lowest in the WC group. The frequency of underlying maternal disease was highest in the WC group. In the first trimester of pregnancy, the PC group often had poor blood sugar control, even at 3 months before pregnancy. Overall, 92.92% of mothers in the WC group and 82.72% of those in the IC group showed good blood sugar control in the third trimester, while only 11.43% of mothers in the PC group had good blood sugar control (HbA1c levels < 6.5%) in the late stages of pregnancy. In the PC group, mean HbA1c decreased from 8.45% in the first trimester to 7.39% in the second trimester.

The comparison of ultrasound findings by trimester among the three groups is shown in Table 2. There were no statistically significant differences in estimated fetal weight, head size, and fetal abdominal circumference in the second trimester across all three groups. However, in the third trimester, the PC group had a greater estimated fetal weight than the WC group (percentile 66.24 ± 30.70 vs. 52.99 ± 27.17; *p* = 0.009). Additionally, the fetal abdominal circumference was significantly different according to glucose control. Comparing the WC and IC groups, estimated fetal weight and abdominal circumference in the third trimester showed no significant differences. There was no significant association between glucose control and amniotic fluid volume.

The comparison of maternal and pregnancy-related outcomes by group is shown in Table 3 and Table 4. Although the admission rates during pregnancy were not significantly different, the reasons for admission varied among the groups: the WC group had a higher incidence of preterm labor and short cervix, while the PC group had a relatively higher number of hospitalizations for PAH or diabetes control. The IC group showed pregnancy-related outcomes similar to those in the WC group. The composite complications during pregnancy among the three groups were significantly different, which was highest in the PC group (*p* = 0.002). Regarding obstetric outcomes, gestational week at delivery and incidence of preterm birth were not significantly different among the three groups. However, the PC group had a significantly higher frequency of shoulder dystocia (44.44% vs. 9.76% vs. 20.00%; *p* = 0.041).

The neonatal outcomes based on HbA1c control are shown in Table 5. The WC and PC groups showed statistically significant differences in neonatal birthweight, hemoglobin, and the incidence of LGA. However, there were no statistically significant differences in other neonatal outcomes among the three groups.

**Figure 2:**
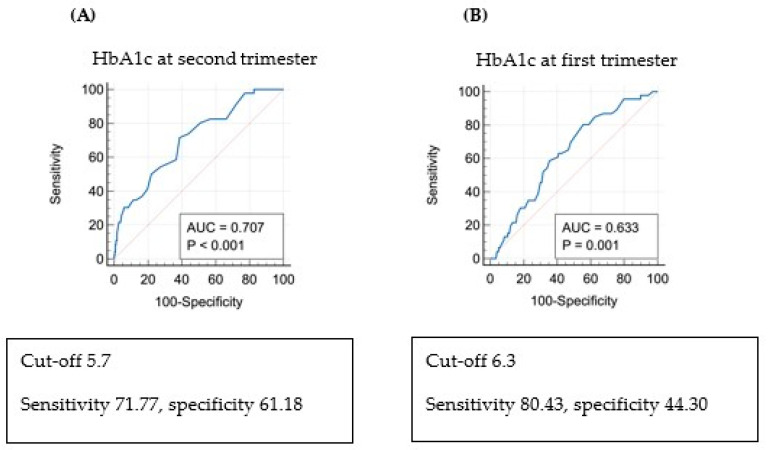
Receiver operating characteristic curve (ROC) and risk of pregnancy-associated hypertension (PAH) via multivariate regression analysis: (**A**) ROC curve: PAH and HbA1c in the second trimester of pregnancy. (**B**) ROC curve: PAH and HbA1c in the first trimester of pregnancy.

The ROC curve between HbA1c levels and PAH in the first and second trimesters is shown in Figure 2. The cut-off value for HbA1c was 6.3% in the first trimester (area under the curve [AUC] 0.633, *p* = 0.001) and 5.7% in the second trimester (AUC 0.707, *p* < 0.001). The analysis of risk factors for PAH using logistic regression (Table 6) revealed that when HbA1c in the second trimester was >5.7%, PAH was 2.9 times more frequent (adjusted OR 2.906; 95% confidence interval [CI]: 1.390–6.075; *p* = 0.005).

**Figure 3:**
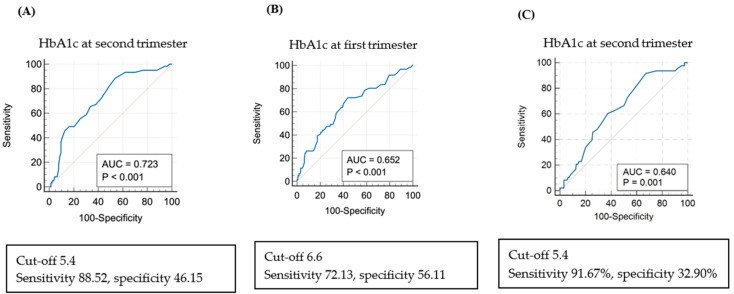
Receiver operating characteristic curve (ROC) and risk of large for gestational age (LGA) via multivariate regression analysis: (**A**) ROC curve: LGA and HbA1c in the second trimester of pregnancy. (**B**) ROC curve: LGA and HbA1c in the first trimester of pregnancy. (**C**) ROC curve for the cut-off value of HbA1c in the second trimester of pregnancy when the estimated fetal weight measured by ultrasound in the third trimester exceeded the 90th percentile.

The analysis of risk factors for LGA using logistic regression revealed that when HbA1c in the second trimester was >6.5%, LGA was 2.966 times more frequent (adjusted OR 2.966; 95% CI: 1.219-7.218-6.075; *p* = 0.017) (Table 7). Figure 3 shows the ROC curve between HbA1c levels and LGA births in the first and second trimesters. The cut-off value for HbA1c was 6.6% in the first trimester (AUC 0.652, *p* < 0.001) and 5.4% in the second trimester (AUC 0.723, *p* < 0.001). When the estimated fetal weight measured by ultrasound in the third trimester exceeded the 90th percentile, the cut-off value for HbA1c in the third trimester was 5.4 (AUC 0.640, *p* = 0.001).

## 4. Discussion

This study demonstrated that HbA1c levels in the first and second trimesters were significantly associated with pregnancy complications, especially PAH and neonatal LGA. However, the IC group versus the WC group did not show a statistically significant difference in these outcomes, which suggests that there is still a chance to improve pregnancy outcomes in women who achieve good glycemic control in the second trimester despite poor glycemic control in the first trimester. In the WC and IC groups, about 93% and 83% of participants, respectively, showed good glycemic control in the third trimester. However, only 11.4% of women in the PC group had good glycemic control in the third trimester. Therefore, glucose control in the first and second trimesters can be a predictive factor for glucose control in the third trimester.

There is a recent randomized clinical trial comparing groups of women diagnosed with diabetes who received immediate treatment and those who did not. In this study, particularly among women with high blood sugar levels before 20 weeks, the group that received immediate treatment showed a modestly lower incidence of adverse neonatal outcomes [14]. Several studies assessed how blood sugar control in early pregnancy, measured by HbA1c at pregnancy confirmation, influences pregnancy outcomes. Mane et al. published a meta-analysis indicating that HbA1c regulation in early pregnancy is a predictor of adverse pregnancy outcomes [6]. This paper reported that mothers with HbA1c < 6.5%, the cut-off value for diagnosing diabetes in the general population, can be at risk of diabetes-related complications, whereas HbA1c ≥ 5.7% in early pregnancy is a strong indication of pregnancy complications. Additionally, in a New Zealand study, the risk of obstetric complications increased with HbA1c > 5.9% in early pregnancy [15]. Other studies also classed individuals with HbA1c > 5.9% in early pregnancy as a high-risk group for diabetes, suggesting that early intervention in this group could reduce the frequency of complications such as pre-eclampsia and preterm birth [16]. Several other studies indicated that glucose regulation in early pregnancy is closely related to pregnancy outcomes and suggested lowering the HbA1c threshold from the conventional cut-off of 6.5% to improve pregnancy outcomes [6,15,17,18,19]. The current study shows that HbA1c levels > 5.7% in the second trimester can predict PAH risk in women with pregestational diabetes, with an AUC of 0.707, sensitivity of 71.77%, and specificity of 61.18%.

A recently published paper reported 76.6% sensitivity and 82.9% specificity for predicting fetal weight through ultrasound performed during pregnancy in mothers with diabetes, indicating ultrasound measurement as a relatively accurate diagnostic method [20]. Another systematic review reported that ultrasound findings are related to maternal and fetal prognoses in women with pregestational type 1 or 2 diabetes [21]. Our study did not show statistically significant differences in estimated fetal weight, abdominal circumference, and amniotic fluid volume measured in the second trimester among the three groups. However, estimated fetal weight and abdominal circumference percentiles by ultrasonography, which was performed in the third trimester, revealed statistically significant differences among the three groups, with percentiles being highest in the PC group. Therefore, inadequate glycemic control during the second trimester may influence the accumulation of adipose tissue in the fetus, which could further impact glucose metabolism. In the second trimester, fetal bodyweight and abdominal circumference are more influenced by genetic factors rather than the effects of blood sugar regulation. In the third rather than second trimester, fetal bodyweight and abdominal circumference are more influenced by maternal blood sugar levels.

This study found no statistically significant differences in height, head circumference, 1 and 5 min Apgar scores, and the composite morbidity of newborns, including NICU admission status, RDS, and congenital anomalies, among the three groups. This is consistent with other studies reporting a low correlation between maternal glucose control and neonatal RDS or hyperbilirubinemia [22,23]. We found similar birthweight in the WC and IC groups, but birthweight was statistically significantly higher in the PC group. Hence, if blood sugar is not well controlled during the second trimester of pregnancy, it affects fetal growth. Additionally, the level of blood sugar control during the second versus first trimester has a greater impact on birthweight. Moreover, SGA was not significantly different among the three groups, although LGA was significantly more frequent in the PC group (51.16%) than in the other two groups (IC: 21.30% and WC: 12.06%) (Table 5). Other studies reported a weak association between glucose levels during pregnancy and SGA [5,23]. Further, among factors causing SGA, maternal blood sugar control has a smaller impact than maternal economic status and lifestyle habits [24]. A logistic regression analysis found that LGA was approximately 3.6 times more frequent in women with HbA1c ≥ 6.5% in the second trimester. Based on the ROC curve analysis, the cut-off value for HbA1c in the second trimester for LGA occurrence was 5.4%. Although this cut-off level is more stringent than that used in other studies, the high incidence of shoulder dystocia in our study highlights the importance of reducing the risk of LGA if such risk reduction can be achieved without marked hypoglycemia, and considering the small physique of Asian women.

The strengths of this study are that it used data from a single country and ethnicity, thereby excluding racial differences. Several studies reported that racial factors significantly influence the prevalence of diabetes and glucose regulation [2,25,26], and bias can be introduced due to heterogeneity in studies involving multiple ethnicities. Therefore, the homogeneity in this study is a strength. Second, instead of comparing HbA1c values in early or late pregnancy alone, we compared the prognosis of pregnancy and newborns by measuring HbA1c serially during the first and second trimesters, using the flow of glucose regulation. Thus, the HbA1c values differed from values measured individually at each stage in several previously published studies.

### Limitations

This study also has some limitations. First, the sample size was small despite involving multiple centers. Second, patient selection bias was possible, as the study targeted mothers diagnosed and regularly followed up at tertiary hospitals. We speculated that the lack of a significant difference in hospitalization rate among the groups and the relatively high rate of hospitalization due to threatened preterm labor in the WC group suggest selection bias. Third, we could not evaluate glucose control levels with other parameters, such as self-monitoring of blood glucose or continuous glucose monitoring (CGM), due to the retrospective study design. Although the American Diabetes Association recommends an HbA1c target of <6.0–6.5% in the first trimester and <6% in the second and third trimesters, ideal target levels during pregnancy remain unclear if the target can be achieved without significant hypoglycemia. This is because HbA1c levels are affected by increased red blood cell turnover, hemodilution, iron deficiency anemia, and other physiologic changes during pregnancy [27]. As CGM was introduced in 2020 for type 1 diabetes in South Korea and, as national insurance has covered (from November 2024) the cost of CGM in women with type 2 diabetes and gestational diabetes requiring insulin, further studies about glycemic control and pregnancy outcomes are needed

## 5. Conclusions

This study compared maternal and neonatal outcomes by dividing mothers with pregestational diabetes into WC, PC, and IC groups, based on serially measured HbA1c values during the first and second trimesters. Glucose regulation in the second trimester had a significant impact on maternal and neonatal prognosis. If glucose regulation was not achieved in the first trimester but was well managed in the second trimester, there would be no difference in prognosis in the PC and IC groups compared to the WC group. Therefore, active intervention to control HbA1c during pregnancy may decrease the risks of PAH and neonatal LGA. Additionally, the cut-off values for the occurrence of PAH and LGA were HbA1c levels of 5.7% and 5.4%, respectively, in the second trimester. Maintenance of HbA1c levels at <5.4% during the second trimester, without significant hypoglycemia, may help to improve pregnancy outcomes in Korean women.

## Figures and Tables

**Table 1:** Maternal characteristics according to glycemic control during the first and second trimesters.

	Well- Controlled (*n* = 141)	Improved- Control (*n* = 108)	Poorly- Controlled (*n* = 43)	*p*-Value ^a^	*p*-Value ^b^	*p*-Value ^c^
Age, years	34.38 ± 3.77	34.05 ± 4.28	33.37 ± 4.60	0.366	0.520	0.149
Parity				0.050	0.116	0.336
Nulliparity	78 (55.32%)	48 (44.44%)	28 (65.12%)			
Type of pregnancy		±		0.408	0.418	0.247
Natural	107 (90.07%)	96 (88.89%)	41 (95.35%)			
ART	14 (9.93%)	12 (11.11%)	2 (4.65%)			
Maternal height, cm	161.09 ± 5.93	161.54 ± 6.07	163.58 ± 6.00	0.059	0.554	0.017 *
Weight before pregnancy, kg	67.02 ± 15.00	70.32 ± 15.07	72.57 ± 13.90	0.062	0.090	0.038 *
BMI before pregnancy, kg/m^2^	25.76 ± 5.08	26.88 ± 5.17	27.02 ± 4.59	0.150	0.088	0.159
Intrapartum BMI, kg/m^2^	30.06 ± 5.78	31.54 ± 6.31	32.57 ± 5.46	0.025 *	0.055	0.012 *
Weight gain during pregnancy, kg	11.26 ± 7.26	12.33 ± 8.28	13.96 ± 7.85	0.134	0.284	0.043 *
Underlying disease	47/141 (33.33%)	17/107(15.89%)	8/43 (18.60%)	0.004 *	0.003 *	0.098
Glucose control type				0.087	0.039 *	0.118
Diet control	10 (7.09%)	0 (0.00%)	2 (4.65%)			
insulin	125 (88.65%)	105 (97.22%)	40 (93.02%)			
Oral agents	5 (3.55%)	1 (0.93%)	0 (0.00%)			
Insulin + oral agents	1 (0.71%)	1 (0.93%)	0 (0.00%)			
HbA1c before pregnancy, %	6.41 ± 0.88	8.44 ± 2.11	8.46 ± 2.68	<0.001 *	<0.001 *	0.005 *
HbA1c in the first trimester, %	5.87 ± 0.42	7.98 ± 1.26	8.45 ± 1.09	<0.001 *	<0.001 *	<0.001 *
HbA1c in the second trimester, %	5.37 ± 0.44	5.78 ± 0.46	7.39 ± 0.86	<0.001 *	<0.001 *	<0.001 *
HbA1c in the third trimester, %	5.64 ± 0.56	6.05 ± 0.61	7.61 ± 1.12	<0.001 *	<0.001 *	<0.001 *
Well-controlled glucose in the third trimester, *n* (%)	105 (92.92)	67 (82.72)	4 (11.43)	<0.001 *	0.048 *	<0.001 *

^a^ *p*-value among the three groups; ^b^ *p*-value between the well-controlled and improved-control groups.; ^c^ *p*-value between the well-controlled and poorly-controlled groups; Underlying diseases were diagnosed before pregnancy and were cases where medication was required: hyperthyroidism, hypothyroidism, thyroid cancer, chronic kidney disease, major depression, and respiratory diseases such as asthma. Well-controlled glucose in the third trimester is defined as HbA1c < 6.5%; ART, artificial reproductive technique; BMI, body mass index; * Statistically significant *p*-values < 0.05 are shown in bold with an asterisk (*). The data are presented as mean ± SD.

**Table 2:** Comparison of estimated fetal weight and biophysical profile values measured through serial ultrasound during pregnancy, according to HbA1c control.

	Well -Controlled (*n* = 141)	Improved-Control (*n* = 108)	Poorly-Controlled (*n* = 43)	*p*-Value ^a^	*p*-Value ^b^	*p*-Value ^c^
At second trimester						
EFW (percentile)	52.53 ± 25.01	55.31 ± 23.42	52.90 ± 27.28	0.710	0.412	0.939
BPD (percentile)	46.76 ± 24.50	48.12 ± 26.91	48.90 ± 31.49	0.935	0.785	0.735
AC (percentile)	47.47 ± 27.04	46.78 ± 24.78	53.63 ± 28.92	0.523	0.892	0.343
At the third trimester						
EFW (percentile)	52.99 ± 27.17	56.49 ± 27.21	66.24 ± 30.70	0.030 *	0.335	0.009 *
BPD (percentile)	49.34 ± 29.64	52.24 ± 30.31	52.84 ± 34.40	0.835	0.604	0.623
AC (percentile)	49.34 ± 34.90	55.17 ± 34.72	70.64 ± 28.78	0.022 *	0.370	0.005 *
AFI before delivery	13.39 ± 4.61	13.31 ± 4.41	14.44 ± 5.99	0.430	0.891	0.325
DP before delivery	4.81 ± 2.11	5.03 ± 1.36	5.11 ± 1.61	0.741	0.556	0.532
Polyhydramnios, *n*	6/129 (4.65%)	2/96 (2.08%)	4/40 (10.00%)	0.129	0.506	0.385
Oligohydramnios, *n*	5/129 (3.88%)	3/95 (3.12%)	2/40 (5.00%)	0.869	1.000	1.000

^a^ *p*-value among the three groups.; ^b^ *p*-value between the well-controlled and improved-control groups.; ^c^ *p*-value between the well-controlled and poorly-controlled groups; AC, abdominal circumference; AFI, amniotic fluid index; BPD, biparietal diameter; DP, deepest pocket of amniotic fluid; EFW, estimated fetal weight. * Statistically significant *p*-values < 0.05 are shown in bold with an asterisk (*). The data are presented as mean ± SD.

**Table 3:** Comparison of maternal outcomes based on HbA1c control during pregnancy.

	Well- Controlled (*n* = 141)	Improved -Control (*n* = 108)	Poorly- Controlled (*n* = 43)	*p*-Value ^a^	*p*-Value ^b^	*p*-Value ^c^
Admission during pregnancy	48 (34.04%)	47 (43.52%)	22 (51.16%)	0.088	0.163	0.065
Diagnosis at admission				0.023 *	0.225	0.023 *
PTL	21/48 (44.68%)	19/47 (41.30%)	4/22 (19.05%)			
PPROM	1/48 (2.13%)	5/47 (10.87%)	0/22 (0.00%)			
IIOC	5/48 (10.64%)	2/47 (4.35%)	1/22 (4.76%)			
PAH	6/48 (12.77%)	5/47 (10.87%)	7/22 (33.33%)			
Uncontrolled DM	6/48 (12.77%)	11/47(23.91%)	8/22 (38.10%)			
ACS use	20/48 (41.67%)	11/45 (24.44%)	3/21 (14.29%)	0.044 *	0.123	0.052
Tocolytic use	23/48 (47.92%)	10/47 (21.28%)	4/22 (18.18%)	0.007 *	0.012 *	0.035 *
PAH	14/141 (9.93%)	18/108 (16.67%)	16/43 (37.21%)	<0.001 *	0.167	<0.001 *
Type of PAH				0.002 *	0.112	<0.001 *
Gestational HTN	3/14 (2.13%)	7/18 (6.48%)	3/16 (6.98%)			
PE, mild	4/14 (2.84%)	4/18 (3.70%)	6/16 (13.95%)			
PE, severe	7/14 (4.96%)	4/18 (3.70%)	5/16 (11.63%)			
Eclampsia	0/14 (0.0%)	3/18 (2.78%)	2/16 (4.65%)			
Infection	5/140 (3.57%)	4/108 (3.70%)	4/43 (9.30%)	0.251	1.000	0.264
Diabetic nephropathy	4/140 (2.86%)	4/108 (3.70%)	3/43 (6.98%)	0.464	0.991	0.437
Diabetic retinopathy	6/140 (4.29%)	4/108 (3.70%)	4/43 (9.30%)	0.322	1.000	0.378
Diabetic neuropathy	3/140 (2.14%)	1/108 (0.93%)	1/43 (2.33%)	0.724	0.806	1.000
Diabetic ketoacidosis	0 (0.0%)	2/108 (1.85%)	2/43 (4.65%)	0.063	0.368	0.084
Composite adverse outcome during ^d^	55/140 (39.29%)	58/108 (53.70%)	29/43 (67.44%)	0.002 *	0.033 *	0.002 *

^a^ *p*-value among the three groups.; ^b^ *p*-value between the well-controlled and improved-control groups.; ^c^ *p*-value between the well-controlled and poorly-controlled groups.; ^d^ Composite adverse outcome during pregnancy defined as any of the following: hospitalization during pregnancy, PAH, diabetes-related retinopathy, nephropathy, neuropathy, ketoacidosis, or infection during pregnancy.; PTL, preterm labor; PPROM, premature preterm rupture of membrane; IIOC, incompetence of cervix; PAH, pregnancy-associated hypertension; ACS, antenatal steroid; PE, preeclampsia; PA, pregnancy age. * Statistically significant *p*-values < 0.05 are shown in bold with an asterisk (*). The data are presented as mean ± SD.

**Table 4:** Comparison of pregnancy-related outcomes based on HbA1c control during pregnancy.

	Well- Controlled (*n* = 141)	Improved -Control (*n* = 108)	Poorly -Controlled (*n* = 43)	*p*-Value ^a^	*p*-Value ^b^	*p*-Value ^c^
Gestational weeks at delivery, weeks	37.63 ± 2.34	37.24 ± 2.50	37.10 ± 1.27	0.253	0.197	0.058
Preterm birth < 37 weeks	25 (17.73%)	28 (25.93%)	13 (30.23%)	0.134	0.159	0.119
Preterm birtht < 34 weeks	6 (4.26%)	8 (7.41%)	0 (0.0%)	0.144	0.428	0.376
Pretern birth < 32 weeks	5 (3.55%)	4 (3.70%)	0 (0.00%)	0.447	0.747	1.000
Mode of delivery				0.440	0.319	0.272
Normal spontaneous delivery	38 (26.95%)	21 (19.44%)	7 (16.28%)			
Assisted vaginal delivery	3 (2.11%)	4 93.70%)	2 (4.55%)			
Cesarean delivery	100 (70.92%)	83 (76.85%)	34 (79.07%)			
FDIU	0 (0.00%)	0 (0.00%)	0 (0.00%)			
Shoulder dystocia	4/41 (9.76%)	5/25 (20.00%)	4/9 (44.44%)	0.041 *	0.420	0.039 *
Postpartum bleeding	2 (1.41%)	4 (3.70%)	2 (4.65%)	0.389	0.454	0.500
Peripartum complications				0.222	0.480	0.137
Chorioamnionitis	2 (1.41%)	1 (0.93%)	0 (0.00%)			
Postpartum endometritis	0	0	0			
Wound infection /dehiscence	0 (0.0%)	0 (0.0%)	1 (2.27%)			
Composite obstetric complications at delivery ^d^	25 (17.73%)	31 (28.70%)	18 (41.86%)	0.004 *	0.057	0.002 *

^a^ *p*-value among the three groups.; ^b^ *p*-value between the well-controlled and improved-control groups.; ^c^ *p*-value between the well-controlled and poorly-controlled groups.; ^d^ Composite obstetric adverse outcome defined as any one of the following: FDIU, preterm birth, shoulder dystocia, chorioamnionitis, postpartum endometritis, wound infection or dehiscence, and postpartum bleeding.; FDIU, fetal demise in utero. * Statistically significant *p*-values < 0.05 are shown in bold with an asterisk (*). The data are presented as mean ± SD.

**Table 5:** Comparison of neonatal outcomes based on HbA1C control during pregnancy.

	Well- Controlled (*n* = 141)	Improved-Controlled (*n* = 108)	Poorly -Controlled (*n* = 43)	*p*-Value ^a^	*p*-Value ^b^	*p*-Value ^c^
Male baby, *n* (%)	66 (46.81)	65 (60.19)	23 (53.49)	0.111	0.049	0.553
Birthweight, g	3105.77 ± 716.18	3129.43 ± 722.65	3478.00 ± 800.09	0.012 *	0.797	0.004 *
LGA, *n* (%)	17 (12.06%)	23 (21.30%)	22 (51.16%)	<0.001 *	0.073	<0.001 *
SGA, *n* (%)	11 (7.80%)	7 (6.48%)	4 (9.30%)	0.827	0.879	1.000
HC, percentile	55.83 ± 44.29	51.08 ± 26.33	58.70 ± 31.79	0.593	0.454	0.753
Height, percentile	54.28 ± 26.64	54.92 ± 23.43	62.95 ± 25.54	0.228	0.879	0.115
Apgar score < 7 at 1 min, *n* (%)	27 (19.29%)	24 (22.22%)	13 (30.23%)	0.316	0.683	0.191
Apgar score < 7 at 5 min, *n* (%)	10 (12.82%)	4 (6.67%)	1 (4.76%)	0.346	0.367	0.514
NICU admission, *n* (%)	48 (34.29%)	45 (43.27%)	21 (50.00%)	0.127	0.195	0.097
UA pH	7.29 ± 0.10	7.29 ± 0.09	7.28 ± 0.09	0.887	0.862	0.704
UA pH < 7.1, *n* (%)	2 (2.06%)	1 (1.49%)	1 (4.00%)	0.758	1.000	1.000
Hemoglobin, g/dL	15.70 ± 2.76	17.07 ± 2.58	17.92 ± 2.45	0.001 *	0.004 *	0.002 *
Glucose, mg/dL	67.31 ± 22.24	68.25 ± 23.62	62.19 ± 23.60	0.379	0.757	0.224
Hypoglycemia	10 (7.58%)	8 (7.92%)	7 (18.92%)	0.092	1.000	0.086
Calcium, mg/dL	8.78 ± 1.48	8.59 ± 1.66	8.73 ± 1.38	0.747	0.455	0.860
Hypocalcemia	12 (11.54%)	12 (15.79%)	4 (13.79%)	0.709	0.544	0.994
Total bilirubin, mg/dL	7.46 ± 3.57	7.97 ± 4.67	7.25 ± 3.95	0.623	0.438	0.776
Hyperbilirubinemia	27 (23.08%)	29 (36.25%)	11 (33.33%)	0.115	0.064	0.332
RDS	16 (11.43%)	19 (18.45%)	6 (14.29%)	0.305	0.175	0.819
Sepsis	6 (4.29%)	2 (1.94%)	1(2.38%)	0.559	0.517	0.916
Cardiomyopathy	1 (0.71%)	1 (0.97%)	1 (2.38%)	0.647	1.000	0.948
Pulmonary HTN	3 (2.14%)	1 (0.97%)	1 (2.38%)	0.746	0.842	1.000
Seizure	2 (1.43%)	0 (0.00%)	0 (0.00%)	0.350	0.613	1.000
Congenital anomaly	17/140 (12.14%)	17/104 (16.35%)	9/42 (21.43%)	0.301	0.453	0.209
Composite morbidity ^d^	49 (35.00%)	47 (45.19%)	22 (52.38%)	0.079	0.139	0.065

^a^ *p*-value among the three groups.; ^b^ *p*-value between the well-controlled and improved-control groups.; ^c^ *p*-value between the well-controlled and poorly-controlled groups.; ^d^ Composite neonatal morbidity defined as the following conditions: NICU admission, Apgar score < 7 at 1 and 5 min, congenital anomaly, umbilical cord pH < 7.1, hypoglycemia (<40 mg/dL), hyperbilirubinemia (>15 mg/dL), hypocalcemia (<7–8 mg/dL), polycythemia (>22 mg/dL), RDS, sepsis, cardiomyopathy, pulmonary hypertension, or seizure.; LGA, large for gestational age; SGA, small for gestational age; HC, head circumference; NICU, neonatal intensive care unit; UA, umbilical artery; RDS, respiratory distress syndrome; HTN, hypertension. * Statistically significant *p*-values < 0.05 are shown in bold with an asterisk (*). The data are presented as mean ± SD.

**Table 6:** Multivariate regression analysis of PAH risk.

	Unadjusted OR	*p*-Value	Adjusted OR ^a^	*p*-Value
Maternal age Pre-pregnancy maternal weight	0.985 (0.913–1.062) 1.041 (1.020–1.062)	0.696 0.000 *	0.999 (0.838–1.190) 1.036 (1.013–1.060)	0.990 0.002 *
Poorly controlled DM (HbA1c > 5.7% in the second trimester)	3.875 (1.976–7.601)	0.000 *	2.906 (1.390–6.075)	0.005 *
Poorly controlled DM (HbA1c > 6.5% in the second trimester)	4.019 (1.954–8.266)	0.000 *	1.713(0.636–4.618)	0.287

^a^ Adjusted for parity, pre-pregnancy BMI, weight at delivery, BMI at delivery, maternal underlying disease, first trimester HbA1c >6.5%, third trimester HbA1c >6.5%.; BMI, body mass index; DM, diabetes mellitus; OR, odds ratio; PAH, pregnancy-associated hypertension. * Statistically significant *p*-values <0.05 are shown in bold with an asterisk (*).

**Table 7:** Multivariate regression analysis of the risk of LGA.

	Unadjusted OR	*p*-Value	Adjusted OR ^a^	*p*-Value
Maternal age Weight gain during pregnancy	0.963 (0.923–1.005) 1.073 (1.044–1.104)	0.081 0.000 *	0.941 (0.863–1.027) 1.105 (0.993–1.229)	0.175 0.183
BMI before pregnancy BMI at delivery	1.055 (1.019–1.092) 1.088 (1.053–1.125)	0.003 * 0.000 *	0.314 (0.066–1.496) 2.685 (0.718–10.013)	0.146 0.142
Poorly controlled DM (HbA1c > 6.5% in the first trimester)	2.761 (1.663–4.586)	0.000 *	2.269 (1.014–5.078)	0.046 *
Poorly controlled DM (HbA1c > 6.5% in the second trimester)	5.474 (2.754–10.880)	0.000 *	2.966 (1.219–7.218)	0.017 *

^a^ Adjusted for parity, pre-pregnancy weight, underlying disease, and weight at delivery.; BMI, body mass index; DM, diabetes mellitus; LGA, large for gestational age; OR, odds ratio. * Statistically significant *p*-values < 0.05 are shown in bold with an asterisk (*).

## Data Availability

The datasets used and/or analyzed during the current study are available from the corresponding author upon reasonable request.

## Abbreviations

Glycosylated hemoglobin, HbA1c; pregnancy-associated hypertension, PAH; receiver operating characteristic, ROC curve; large for gestational age, LGA; respiratory distress syndrome, RDS; body mass index, BMI; fetal death in utero, FDIU; small for gestational age, SGA; neonatal intensive care unit, NICU; odds ratio, OR.
